# Is the Epidemiology of Alkhurma Hemorrhagic Fever Changing? : A Three-Year Overview in Saudi Arabia

**DOI:** 10.1371/journal.pone.0085564

**Published:** 2014-02-06

**Authors:** Ziad A. Memish, Shamsudeen F. Fagbo, Ahmed Osman Ali, Rafat AlHakeem, Fathelrhman M. Elnagi, Elijah A. Bamgboye

**Affiliations:** 1 Public Health Directorate, Ministry of Health, Riyadh, Kingdom of Saudi Arabia; 2 Ministry of Health, Najran, Kingdom of Saudi Arabia; 3 College of Medicine, Al-Faisal University, Riyadh, Kingdom of Saudi Arabia; 4 Department of Medical Statistics and Epidemiology, University of Ibadan, Ibadan, Nigeria; Metabiota, United States of America

## Abstract

**Background:**

The epidemiology of Alkhurma hemorrhagic fever disease is yet to be fully understood since the virus was isolated in 1994 in the Kingdom of Saudi Arabia.

**Setting:**

Preventive Medicine department, Ministry of Health, Kingdom of Saudi Arabia.

**Design:**

Retrospective analysis of all laboratory confirmed cases of Alkhurma hemorrhagic fever disease collected through active and passive surveillance from 1^st^-January 2009 to December, 31, 2011.

**Results:**

Alkhurma hemorrhagic fever (AHFV) disease increased from 59 cases in 2009 to 93 cases in 2011. Cases are being discovered outside of the region where it was initially diagnosed in Saudi Arabia. About a third of cases had no direct contact with animals or its products. Almost all cases had gastro-intestinal symptoms. Case fatality rate was less than 1%.

**Conclusions:**

Findings in this study showed the mode of transmission of AHFV virus may not be limited to direct contact with animals or its products. Gastro-intestinal symptoms were not previously documented. Observed low case fatality rate contradicted earlier reports. Close monitoring of the epidemiology of AHFV is recommended to aid appropriate diagnosis. Housewives are advised to wear gloves when handling animals and animal products as a preventive measure.

## Introduction

### Background

Alkhurma hemorrhagic fever virus (AHFV) first emerged in 1995 when it was isolated at Soliman Fakeeh Hospital from samples taken from a butcher with fatal hemorrhagic fever. Archived samples in the same hospital from patients with similar clinical presentation also yielded the same virus [1], now named Alkhurma hemorrhagic fever virus (AHFV) [2] . It has since been confirmed to be a tick -borne flavivirus with close similarity with the Kyasanur Forest disease virus found in India [3]–[4]. Other tick borne flaviviruses include Omsk Hemorrhagic fever virus and Tick-borne encephalitis viruses [4] although AHFV is the first tick-borne flavivirus linked to laboratory confirmed clinical illness in the Arabian Peninsula [2]. Initially, the clinical disease caused by AHFV did not receive much attention and its epidemiology was not well comprehended: one early publication on clinical cases not only failed to consider the notorious cross reacting properties of flavivirus antibodies in patients recovering from flavivirus infections but also misinterpreted lack of tick bite histories as evidence against AHFV being tick borne [5] and over-interpreted mosquito bite histories to suggest that the virus was mosquito-borne.

Earlier studies detecting and/or isolating AHFV in soft (*Ornithodoros savigni*) and hard (*Hyalomma dromedarii*) ticks collected from camel and sheep clearly suggested a role for these vectors in AHFV transmission [6]–[7]. Additionally, AHFV patients have been epidemiologically linked to exposure to blood of slaughtered animals, consumption of raw milk as well as tick bites [3]. A Mosquito-borne transmission has been suggested in the literature [5], [8] but this remains unsubstantiated as also observed by other authors [9]. Recently, the detection of AHFV infection in 2 tourists returning to Italy from a visit to Southern Egypt may suggest that AHFV may exist outside the Arabian Peninsula albeit undetected or misdiagnosed. These 2 patients were tested and found to have cross reactive antibodies to both West Nile fever and DEN viruses [10], [11]. This report renews the discourse as regards the origin of AHFV as well as other tick borne flaviviruses [4]: Previously published molecular data has indicated tick borne flaviviruses originated from Africa [12]. However, it is important to increase awareness and effective surveillance to detect cases of AHFV infection in geographically diverse areas of the world. Thus a regular review of all confirmed AHFV infections will enrich the scientific literature of the epidemiology of the disease its causes.

### Objectives

The objective of this report therefore is to describe all cases of Alkhurma disease seen in Saudi Arabia over a 3-year period (2009–2011) along the three basic epidemiological principles- person, place and time.

## Methods

### Study design

We retrospectively analysed all cases of AHFV infection, laboratory confirmed by RT-PCR and reported over a period of three years (2009–2011) in Saudi Arabia. Simple descriptive statistical tools in the SPSS software package were used to summarise and present data [13].

### Ethics Statement

The study did not require an ethical approval because it was a retrospective analysis of data from the Alkhurma surveillance system. The database is hosted at the Department of Preventive Medicine, Ministry of Health, Riyadh, Saudi Arabia under the sole authority of the Deputy Minister for Public Health. All data analyzed were de-identified for anonymity before it was made available for data analysis.

### Alkhurma case definition

A suspected case of AHFV infection is defined as someone who presents with an acute onset of fever and flu-like illness with or without hemorrhagic signs or symptoms, hepatomegaly or elevated liver enzymes and encephalopathy. A suspected case residing in the Kingdom of Saudi Arabia (KSA) with a recent history of contact with animals; including their products (milk, meat, fetuses, aborts and carcasses), with evidence of leucopenia and or thrombocytopenia and or elevated LFTs was considered a probable case. A confirmed case is Alkhurma disease was comprised of any suspected or probable case whose laboratory investigation using PCR is positive.

### Laboratory method

The laboratory test used was the Real -Time Reverse -Transcriptase PCR based on the commercial kit made by TIB Molbiol and optimized to work with Roche's Light Cycler 2.0 [14]. Positive cases of Alkhurma disease were those that were real time RT-PCR positive.

### Procedure

The detection of Alkhurma virus was undertaken using the Roche MagNA pure instrument (Roche) .200 µl of each sample was added to sterile plates or 2 ml tubes . Reaction reagents were then loaded and checked before running the samples according to the manufacturer's instructions for nucleic acid extraction in specimen area.

#### Reverse transcription (RT-PCR) Step

The purpose of this step is to produce the complementary DNA (c DNA) in order to use it in the next step as a template. Viral RNA was performed in the extraction stage with Transcriptor First Strand cDNA Synthesis Kit (Roche)

The RT reaction mixture was prepared by Transcriptor First Strand cDNA Synthesis Kit, Following this, 2 µl random hexamers primers, 5 µl (1 to 100 ng) from extracted RNA and 6 µl of PCR grade water was added to give 13 µl in total volume. This is followed by incubation at 65°C for 10 min in order to ensure denaturation of RNA secondary structures. After this, the following reaction was added to each sample, 4 µl of transcriptor RT reaction buffer, 0.5 µl of protector Rnase inhibitor, 2 µl of dNTP mix and 0.5 µl of transcriptor reverse transcriptase to give 7 µl of total volume. All tubes with total volume of 20 µl of RT-PCR reaction mix was subjected to RT-PCR amplification step by following this thermal profile, 25°C for 10 min, 55°C for 30 min and finished with 85°C for 5 min and then held at 4°C.

#### Real Time PCR Mixture reaction (Amplification reaction)

LightMix detection kit was used for screening suspected samples with Alkhruma virus by following the manual instruction protocol. The sensitivity detection limit (10 Copies/5 µl) of this two-step RT-PCR for the detection of Alkhurma virus is usually obtained at CT value (35–37).

### Alkhurma surveillance system

AHFV is one of the nationally notifiable diseases in KSA and an effective surveillance system for it was established by the Ministry of Health. The system requires an immediate reporting of all suspected cases of Alkhurma according to the criteria of the case definition using standardized forms. There are 3 standardized forms used to capture all relevant clinical, epidemiological, and laboratory data. These are a suspect case form, an epidemiological investigation form, and a laboratory requisition form. The data flow starts from the hospitals and health centers (whether governmental or private) to the desk officer in charge of viral hemorrhagic fevers at the directorate of health affairs in each region. Subsequently, the desk officer sends the report to the directorate of infectious diseases at the preventive medicine department at the ministry of health either by fax, telephone or e-mail. The desk officer in charge of AHFV checks the reports for completeness and accuracy before entering them into the database system. The desk officer at the ministry of health prepares weekly, monthly and quarterly reports for the higher directors at the ministry of health and sends them as feedback to the regions and health units.

### Statistical Analysis

The initial data, captured in Microsoft Excel, was transferred to SPSS version 15.0 for statistical analysis [13]. Frequency distribution tables and summary statistics such as proportions and means were used for summarizing categorical and continuous variables, respectively. The patient's occupation which was considered to be a very important variable was classified into five categories. The first category were those who have direct contact with animals such as butchers, shepherds, cooks, abattoir workers, poultry farmers and others involved in the livestock industry. Other cases who reported direct contact with animals apart from their primary jobs such as housewives, who take care of livestock in their homes, were classified into the same group. The second category consisted of other housewives and maids who process meat from potentially infected animals prior to consumption. Cases of **AHFV** in a variety of blue collar jobs including barbers, carpenters, guards, soldiers, tailors and drivers were classified in the third category. The fourth group was comprised of white collar workers such as employers, teachers, office workers, students, nurses and doctors. It should be noted that the only cases in this category were a doctor and a nurse. The last group was those without defined jobs.

## Results

### Epidemiology

**Figure 1:**
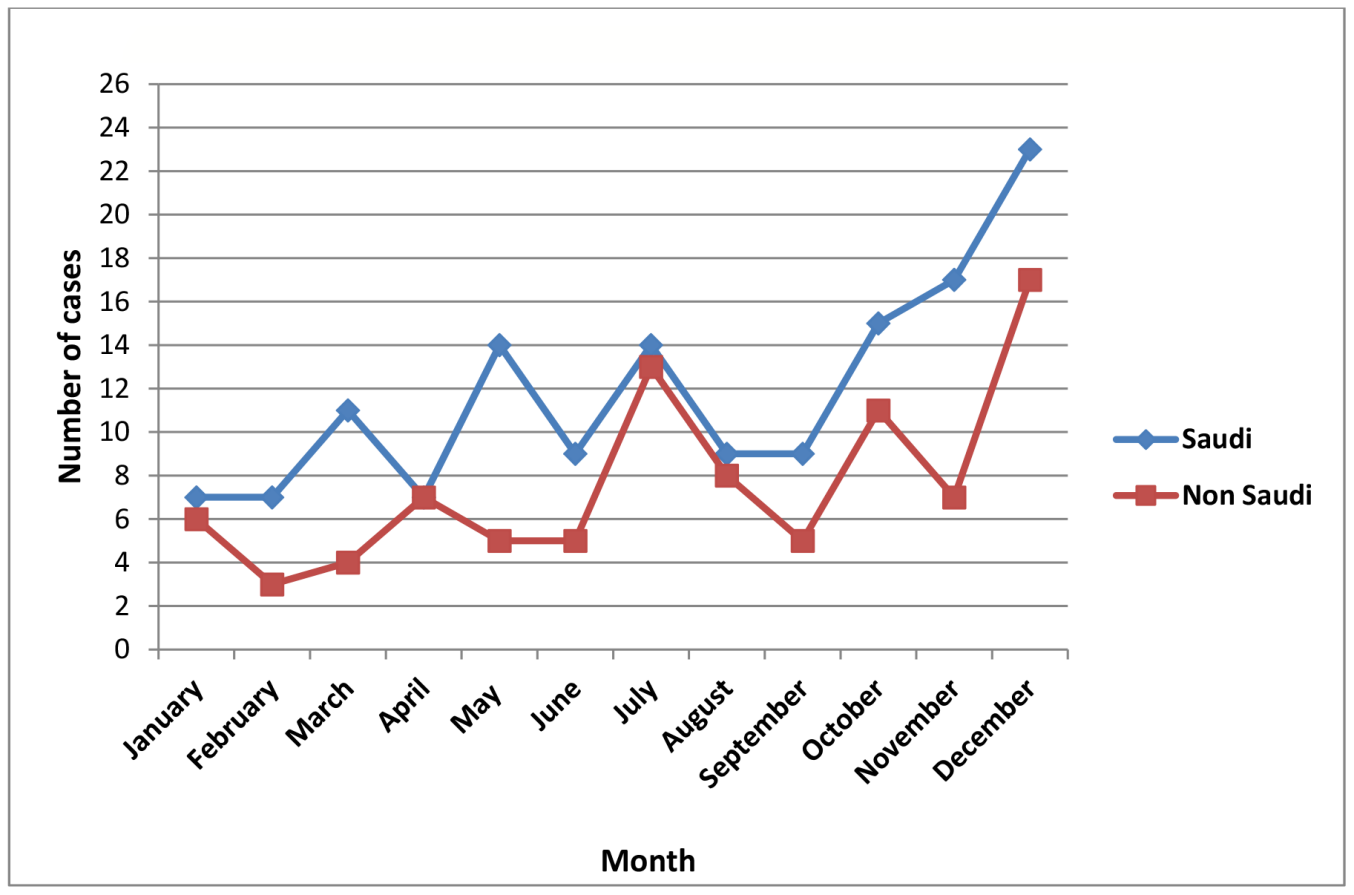
The monthly distribution of Alkhurma disease over a three year period in Saudi Arabia (2009–2011).

Table 1 shows the yearly and provincial distribution by province of all 233 confirmed cases of **AHFV** reported during the three-year period from 2009 to 2011. The lowest frequency of 59(25%) was reported in 2009, increasing to 81(34.8%) in 2010 and 93(39.9%) in 2011. The monthly distribution of the cases shown in Figure 1 revealed it peaks in July and December. The lowest number of cases was reported in February although there were relatively few cases between January and June and August and September.

**Table 1:** The regional distribution of Alkhurma disease in Saudi Arabia- 2009–2011.

Year	Region					
Nationality	Jeddah	Najran	Jizan	Makkah	Taif	Total
2009						
Saudi	3(7.1)	39(92.9)	0	0	0	42(100%)
Non-Saudi	1(5.9)	16(94.1)	0	0	0	17(100%)
Total	4(6.8)	55(93.2)	0	0	0	59(100%)
2010						
Saudi	4(10.0)	33(82.5)	1(2.5)	2(5.0)	0	40(100%)
Non-Saudi	10(24.4)	28(68.3)	0	3(7.3)	0	41(100%)
Total	14(17.3)	61(75.3)	1(1.2)	5(6.2)	0	81(100%)
2011						
Saudi	6(10.0)	48(80.0)	0	5(8.3)	1(1.7)	60(100%)
Non-Saudi	4(12.1)	27(81.8)	0	2(6.1)	0	33(100%)
Total	10(10.8)	75(80.1)	0	7(7.5)	1(1.1)	93(100%)

The age distribution of the patients shown in Table 2 revealed that less than 5% were below the age of 15 years and about a third were 45 years and above. More than 45% of AHFV infected persons were between 25 and 45 years. The youngest was a child of 3 years and the oldest was a man of 85 years. The mean age of all of the cases was 38.8 years (SD = 16.7 years) .The Saudis on the average were older (38.9 years, SD = 17.5 years) than the non-Saudis (33. 3years, SD = 14.8 years) .The females were about 42% of cases showing a slightly male preponderance.

**Table 2:** Some Demographic Characteristics of Patients with Alkhurma disease in Saudi Arabia (2009–2011).

	Nationality		
Variables	Saudi	Non-Saudi	All
**Age group(Panel 1)**			
0–1	1(0.7)	2(2.2)	3(1.3)
05–14	7(4.9)	3(3.3)	10(4.3)
15–24	22(15.5)	21(23.1)	43(18.5)
25–34	32(22.5)	29(31.9)	61(26.2)
35–44	34(23.9)	15(16.5)	49(21.0)
45–54	18(12.7)	10(11.0)	28(13.0)
55+	28(19.7)	11(12.1)	39(16.7)
**Gender (Panel 2)**			
Male	75(52.8)	22(24.2)	97(41.6)
Female	67(47.2)	69(75.8)	136(58.4)
**Occupational group (Panel 3)**			
Direct contact animals jobs	44(31.0)	52(57.1)	96(41.2)
Housewives and helpers	44(31.0)	16(17.6)	60(25.8)
Technicians	7(4.9)	7(7.7)	14(6.0)
Office jobs	38(26.8)	15(16.5)	53(22.7)
No defined jobs	9(6.3)	1(1.1)	10(4.3)
**Regions (Panel 4)**			
Jeddah	13(9.2)	15(16.5)	28(12.0)
Jizan	1(0.7)	0(0.0)	1(0.4)
Makkah	7(5.6)	5(5.5)	12(5.6)
Najran	120(84.5)	71(78.0)	191(82.0)
Taif	1(0.7)	0(0.0)	1(0.4)
**Total**	**142(100%)**	**91(100%)**	**233(100%)**

Panel 3, Table 2 shows that about 42% of all the cases in the three years had occupations that are related to the direct handling of animals like butchers, shepherds, abattoir workers and others involved in the livestock industry. This category of cases was constituted of 31% of Saudi cases and 57% of the non-Saudis cases. The category comprised of housewives and their maids constituted about a quarter of all cases and accounted for nearly 31% of all Saudis and about 18% of non-Saudis. They were the second largest group of these AHFV cases. The third largest group that constituted about 23% of all cases were persons categorised into the group comprised of white collar jobs like teachers, students, and government employees. This group also constituted about 27% of the Saudi cases but only 17% of the Non-Saudi cases. The technicians contained 6% of the AHFV cases.

The last panel (4) in Table 2 shows that about 82% of all cases came from Najran, with a slightly higher proportion of the Saudi cases (85%) than in the non-Saudi cases (78%). One case each was reported from Taif and Jizan provinces. Jeddah accounted for 12% of all cases reported nationwide and the proportion among non-Saudi cases (17%) was higher than the Saudi cases (9%). Almost 5% of the cases were reported from Makkah with equal proportional distribution among Saudi cases and non-Saudi cases.

### Clinical Signs and Symptoms

The commonest signs and symptoms were fever reported by about 96% of all cases with more than 90% having body temperatures above 37.0°C and about 60% had malaise while 40% reported chills. Blood pressure data was available for about 30% of the cases under review and less than 5% had either systolic or diastolic hypertension. About three quarters of the 88 cases with data on pulse rate had normal readings and one case had a high value and the remaining had low values. Also, 25% of the 84 cases with data on respiratory rate data had low readings and about 16% had high readings.

Panel 1 of Table 3 shows that the commonest hemorrhagic manifestations were purpura and epitasis (although reported by only about 5% of the cases) while the least was menorrhagia (2.2%). Pain disorders had headache as the commonest reported by about 60% of cases followed by myalgia (44%) and the least reported was retro-orbital pain by about 16% as shown in panel 2 of Table 3. Gastro-intestinal symptoms were reported by about 99% of all cases. Anorexia (62%), nausea (60%) and vomiting (53%) were the 3 leading gastro-intestinal- symptoms while diarrhea (12%) was the least reported. Less than 10% of the patients reported any symptoms of the central nervous system. Panel 4 of Table 3 showed that the commonest symptoms were disorientation (5.4%) hallucination (4.3%) and convulsion 4.3%).

**Table 3:** Clinical Signs and Symptoms of Patients with Laboratory-Confirmed AHFV infected patients, 2009–2011.

Panels	Symptoms	Frequency	Percent
1	**Bleeding disorders**		
	Epitasis	12	5.2
	Gingival-bleeding	7	3.0
	Petechiae	10	4.3
	Echymosis	10	4.3
	Purpura	12	5.2
	Haematemesis	10	4.3
	Malena	10	4.3
	Rectal bleeding	10	4.3
	Menorrhagia	5	2.1
2	**Pain disorder**		
	Headache	153	65.7
	Retro-orbital pain	37	15.9
	Myalgia	137	58.9
	Arthralgia	102	43.8
	Backache	82	35.2
3	**Gastro-Intestinal Symptoms**		
	Anorexia	145	62.2
	Nausea	140	60.1
	Diarrhea	73	31.3
	Abdominal-pain	93	39.9
	Vomiting	123	52.8
4	**Nervous System**		
	Confusion	7	3.0
	Disorientation	12	5.2
	Hallucination	10	4.3
	Coma	3	1.3
	Convulsion	4	1.7
	Neck rigidity	3	1.3
	Photophobia	3	1.3
	Hemiparesis	3	1.3
5.	**Others**		
	Fever	224	95.6
	Body Temp. >37.0°C	210	90.1
	Chills	93	39.9
	Malaise	137	58.9
	Skin rash	30	12.9

The study also revealed that there were only 2 deaths and these occurred in 2010, no deaths were recorded either in 2009 or 2011. This gave an overall case fatality as 0.43 percent.

## Discussion

The findings in this study have shown that Alkhurma hemorrhagic fever is steadily on the increase in Saudi Arabia and is gradually being reported beyond the geographic locality where it was originally discovered. This corroborates similar findings reported in the literature [15] and this may indicate that the virus is being discovered outside of the area where the initial disease was described. It could also be that there is an increase in awareness of the disease, surveillance and improved laboratory diagnostic capabilities elsewhere [16].

Earlier in its history, the epidemiology of AHFV was described as having a case fatality rate of over 25%. An earlier study suggested this high figure might have been due to earlier laboratory confirmed cases being biased towards detecting severe cases [2]. The much lower case fatality of less than 0.5% found among a large number of cases in the present study supported this earlier position [2]. However, there speculations that the observed low case fatality may also be related to possible changes in the pathogenicity of the AHFV strains causing more recent infections [17]. In addition, more rapid diagnosis, likely development of herd immunity in the population and aggressive symptomatic management may be contributing to the reduced case fatality of AHFV [2], [17]–[19]. Further studies are needed to explore any changes in the pathogenicity of the AHFV strain causing more recent infections.

Our observation that a significant proportion of the AHFV cases in this study had direct contact with animals is indeed consistent with past research findings that have associated direct animal contact with AHFV incidence, albeit in a non-causal manner [20]. The high frequency of housewives also corroborates previous studies that epidemiologically linked AHFV infected housewives in Najran with histories of tick bites [7]. However that as much as a third of cases had no jobs directly related to animal handling or its potentially infected products might just suggest there could be other modes of transmission of the virus. Also, the good proportion of women of more than 40% observed over these three years can be explained by the burden of taking care of livestock often kept within the Saudi compounds borne by these women which could have increased their risk of exposure to tick bites [5], [21]. The vast majority of non- Saudis do not keep animals where they live because their residences are usually not suitable for keeping animals and so this could have limited their direct contact with animals. This might also be a likely explanation for the low proportion of female non-Saudis among cases of the AHFV found in this study.

Of all clinical symptoms observed in the AHFV infected patients in our study, gastro intestinal symptoms such as anorexia, nausea, abdominal pain and vomiting were present in almost all the patients. This was followed by some form of pain in about 90% of AHFV infected cases. Our study also noted that hemorrhagic manifestations such as epitasix occurred in less than 5% of cases. On the other hand, CNS involvement, manifesting as disorientation, hallucination and convulsions, occurred in less than 30% of the patients. These data should be of value in the clinical diagnosis, case definition and management of acute AHFV infections [2].

Our study showed that a distinctively high proportion of cases were reported by the Najran region, an agricultural area with emphasis on livestock farming might be the consequences of a higher exposure to direct contact with animals [7], [13], [20]–[21]. Also, it seemed enhanced surveillance including case investigation in the Najran region may explain the high number of reported cases from this region compared to other regions like Makkah, Jizan, Taif and Asir which have similar agricultural conditions. Also, the presence of putative tick vectors in the region and a higher exposure of females to animals and animal products as housewives could have contributed to the high number of female cases [7], [19]–[22]. However, the absence of a history of tick bite does not rule out tick exposure because tick vectors have been observed in the past and recently in almost all parts of KSA regions that are presently reporting AHFV cases. It can also not be ruled out that transmission of the virus is occurring elsewhere within the country but remains undetected. The singular and first ever case reported from Turbah in the Taif region was fatal. It was diagnosed in a health facility in Jeddah and initially thought to be a case of Dengue infection, and so other similar cases from that region might have been missed or misdiagnosed as Dengue or Brucellosis [2], [5]. Although there is no data on the sensitivity or specificity of PCR assay used to confirm AHFV cases in this study, published data showed that the PCR is sensitive in detecting active disease [22], [23]. To enhance surveillance and address the issue of misdiagnosis and under-reporting, the Ministry of Health in KSA has produced a comprehensive set of guidelines for the diagnosis, management, reporting and prevention of AHFV infection [2]. The guidelines also emphasized the immediate implementation of health education programs within the community in order to prevent transmission especially in communities thought to be at high risk of AHFV infection AHFV [22].

## Conclusion

The high proportion of housewives and maids infected which have also been explained by their possible exposure to this virus while preparing food with meat from possibly infected animals has a policy implication. This could inform the formulation of a policy for targeted public health awareness campaign to stress the need to wear protective equipment including appropriate gloves while cutting and processing meat from freshly slaughtered animals. Also, the mode of transmission of AHFV virus may not be limited to direct contact with animals or its products. And therefore close monitoring of the epidemiology of AHFV is recommended to aid appropriate diagnosis. Gastro-intestinal symptoms were not previously documented and this information should improve clinical diagnosis. Observed low case fatality that is much lower than those of earlier reports should encourage improved clinical management of cases.
